# Surgical Results of Triamcinolone Assisted Pars Plana Vitrectomy Combined with Phacoemulsification in Diabetic Patients

**DOI:** 10.2174/1874364100802010005

**Published:** 2008-01-30

**Authors:** Hakki Birinci

**Affiliations:** Department of Ophthalmology, Medical Faculty, University of Ondokuz Mayıs, Turkey

**Keywords:** Phacoemulsification, triamcinolone assisted vitrectomy, intraocular lens implantation, proliferative diabetic retinopathy.

## Abstract

**Purpose:**

It is aimed to evaluate the technical feasibility, safety, outcome, and incidence of complications after clear corneal phacoemulsification with foldable intraocular lens implantation (IOL) and triamcinolone assisted pars plana vitrectomy in patients with proliferative diabetic retinopathy.

**Methods:**

The results of combined phacoemulsification, in the capsular bag foldable intraocular lens implantation and triamcinolone assisted pars plana vitrectomy in 75 eyes of 72 patients, were retrospectively evaluated. Surgery was performed using general anesthesia in 9 cases, and monitored retrobulbar block in 66 cases. In all cases, phacoemulsification with clear corneal incision and foldable acrylic IOL implantation were performed before vitreoretinal surgery. The main investigation points were preoperative and postoperative best corrected visual acuity (BCVA), and intraoperative and postoperative complications.

**Results:**

The mean age at surgery was 62.07 ± 9.51 years (range 22 to 78 years) and the postoperative follow-up time was 17.17 ± 7.25 months. All eyes had clinically significant cataract that interfered with visualization of the retina preoperatively. All eyes had proliferative diabetic retinopathy. Postoperatively, visual acuity improved in 65 eyes (86.7%), was unchanged in 9 eyes (12.0%), and decreased in 1 eye (1.3%). The most postoperative complications consisted of a mild iritis in 8 eyes (10.7%), recurrent vitreous hemorrhage in 8 eyes (10.7%), posterior capsule opacification in 7 eyes (9.3%), transient intraocular pressure increase in 6 (8.0%), iatrogenic retinal tear in 6 (8.0%), epiretinal membrane in 5 (6.7%).

**Conclusion:**

The results of combined phacoemulsification in the capsular bag foldable IOL implantation and triamcinolone assisted pars plana vitrectomy show that visual acuity outcomes are generally favorable and complications are acceptable in diabetic patients. Visual results and complications depend primarily on the underlying posterior segment pathology. The use of triamcinolone acetonide may simplify surgery and decreases the postoperative inflammation.

## INTRODUCTION

Cataract often coexists with pathologies at the vitreous body. Even if cataract is not significant at the time of vitrectomy, it can progress postoperatively. After pars plana vitrectomy (PPV), cataract development is common (75%). Several authors report difficulties in performing cataract extraction after vitrectomy because of insufficient vitreous support. Lens opacities interfere with appropriate visualization of the vitreous chamber and the eye fundus, which makes performing vitreoretinal procedure difficult. These difficulties led to the idea of performing cataract extraction at the time of vitrectomy. For this reason, in such patients, a combined procedure of PPV and cataract extraction can be considered. The results of combined surgery have been reported many times; however, the results are controversial [1-4].

During PPV it is essential to visualize the residual vitreous cortex and posterior hyaloid. Triamcinolone acetonide (TA) is a water-insoluble steroid and has strong anti-inflamatory  and  antiangiogenic  activity   [5,6].  Intravitreal injection of TA during PPV appeared to aid visualization of the vitreous and posterior hyaloid.

In this study, we aimed to evaluate the technical feasibility, safety, outcome, and incidence of complications after clear corneal phacoemulsification with foldable intraocular lens implantation and triamcinolone-assisted pars plana vitrectomy in the patients with proliferative diabetic patients.

## MATERIALS AND METHODS

The results of combined phacoemulsification, in the capsular bag foldable IOL implantation and triamcinolone assisted pars plana vitrectomy in 75 eyes of 72 patients, were retrospectively evaluated. All patients had clinically significant lens opacities and vitreoretinal pathology requiring pars plana vitrectomy. Patients had surgery by the same surgeon (H.B.) at the department of Ophthalmology, University of Ondokuz May s, between May 2004 and July 2007.

All eyes had proliferative diabetic retinopathy. Typical indications for vitrectomy are recurrent or non-clearing vitreous hemorrhage, tractional retinal detachment and tractional macular edema. The cases of combined tractional-rhegmatogenous retinal detachment were excluded. Preoperative clinical data included the patient’s age, sex, underlying vitreous or retinal disease, and preoperative visual acuity. Keratometry, axial length measurements, B-scan ultrasonographic evaluation and intraocular lens calculation were performed in the eye before surgery. If this not possible, the data were taken from the fellow eye. Intraocular lens calculation was performed using the Binkhorst formula.

Preoperatively, pupils were dilated with drops 1% cyclopentolate hydrochloride, 0.03% flurbiprofen, and 1% tropicamide, three times, 1drop every 10 minutes. Surgery was performed using general endotracheal anesthesia in 9 cases, and monitored retrobulbar block in 66 cases.

The cataract was extracted firstly. A 3.2 mm wide and 1.5 to mm long clear corneal tunnel was created at the superior limbus. A 5.0 to 6.0 mm curvilinear capsulorhexis was completed, Bimanual phacoemulsification and cortex removal were performed. The anterior chamber and capsular bag were filled with sodium hyaluronate (Healon GV) and a foldable acrylic IOL (Sensar AR40^®^, Allergan Surgical)) was inserted into the capsular bag. The visco-elastic material was then removed by irrigation/aspiration. The incision was closed with a single 10-0 nylon suture before vitrectomy.

A standard 3-port pars plana vitrectomy was performed using a 20 gauge vitreous cutter and handheld light pipe under a noncontact wide-angle viewing system (BIOM, Oculus). Sclerotomies were placed 3.5 mm posterior to the limbus in to superotemporal, superonasal, and inferotemporal quadrants. The infusion cannula was sutured in the inferotemporal sclerotomy site. After core vitrectomy, 0.1-0.2 ml triamsinolone acetonide suspension was injected mid-vitreous cavity. The triamcinolone granules were trapped in the gel structure of the residual vitreous cortex. After this procedure, residual vitreous cortex was typically seen on the retina as either diffuse membrane or small islands. These vitreous islands were there-after removed by surgical forceps or a silicon-tipped needle. Intraocular scissors, forceps, and picks were necessarily used to elevate and section membrane if areas of significant preretinal proliferation were encountered. Hemostasis was obtained with the use of the diathermy of the tissue manipulator. After posterior hyaloid separation and removal of residual vitreous cortex, the resection of fibrous tissue and endolaser photocoagulation were performed when needed. Thereafter, we attempted to wash out the residual triamcinolone granules. Fluid/air exchange was performed in 9 eyes (12.0%), silicone oil tamponade in 6 eyes (8.0%), and 20% sulphur hexafluoride (SF6) tamponade in 2 eyes (2.7%). As a last step in the operation, 0.1 ml (4 mg) triamsinolone acetonide was injected into the vitreous cavity of the patients without any tamponade. Sclerotomies and conjunctiva were sutured, and subconjunctival gentamicin sulfate (20 mg) and dexamethasone sodium phosphate (4 mg) were administered.

Postoperative data included Snellen visual acuity, operative and postoperative complications, intraocular pressure, anterior and posterior segment evaluations. Patients were controlled postoperative first week, first month, and every two months. Major outcomes were preoperative and postoperative best corrected visual acuity. Visual improvement was defined as an increase of more than 2 lines on the Snellen acuity chart. Changes from hand movements to counting fingers and from counting fingers to 0.1 were also considered visual improvement.

## RESULTS

The study sample included 75 eyes of 72 patients, 42 women and 29 men. The mean age at surgery was 62.07 ± 9.51 years (range 22 to 78 years) and the postoperative follow-up time 17.17 ± 7.25 months (range 3 to 39 months). All eyes had clinically significant cataract that interfere with visualization of the retina preoperatively.

All corneas remained clear during cataract surgery and pars plana vitrectomy, and throughout the postoperative period. The posterior capsule remained intact in each case and intraocular lenses were implanted in the capsular bag in all cases. Adjunctive vitreoretinal surgery included argon laser photocoagulation, epiretinal membrane peeling, internal limitan membrane peeling, air-fluid exchange in required cases.

**Fig. (1):**
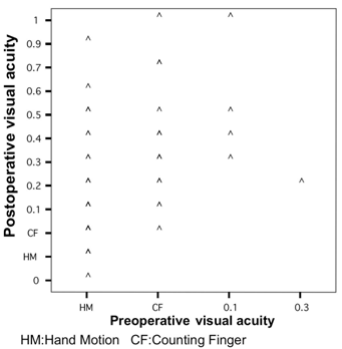
Preoperative and final postoperative visual acuities in the combined surgery.

This study showed an important improvement in visual acuity (Fig. **1**). That is, postoperatively, visual acuity improved in 65 eyes (86.7%), unchanged in 9 eyes (12.0%), and decreased in 1 eye (1.3%). Table **1** describes the best corrected visual acuity outcomes.

The final visual acuity correlated with the vitreoretinal pathology. The greater improvements are seen in the patients with vitreous hemorrhage.

There were few postoperative complications. Intraoperative and postoperative complications in the combined surgery are shown in Table **2**.

Significant recurrent vitreous hemorrhage developed in 8 eyes (10.7%), 4 eyes (5.3%) required surgical evacuation, and 4 eyes (5.3%) cleared spontaneously. Eight eyes (10.7%) had a mild iritis but was controlled and resolved with use of topical steroids in few days. None of the eyes developed anterior chamber fibrin exudation. Posterior capsule opacification occurred in 7 eyes (9.3%). Nd:YAG capsulotomy was performed in 5 eyes (6.7%) because deterioration in postoperative visual acuity. In 6 eyes (8.0%), IOP increased temporarily for a few days after surgery; all were controlled with topical antiglaucomatous medication. Retinal tear occurred in 6 eyes during vitrectomy, which was treated by intraoperative photocoagulation. Silicone oil was used in these eyes after air-fluid exchange. Mild corneal edema developed in 5 eyes (6.7%), and resolved with using topical steroid and 5% NaCl in a few days. Two eyes (2.7%) developed postoperative hyphema; however, none required further surgical evacuation and cleared spontaneously. Rhegmatogenous retinal detachment developed in 2 eyes (2.7%) postoperatively and the retina remained attached after silicone oil injection.

Optic atrophy in various degrees was detected in 12 eyes (16.0%) postoperatively, and poor visual outcome occurred in these eyes.

One eye (1.3%) developed phthisis. This eye had included severe proliferative diabetic retinopathy, vitreous hemorrhage and massive tractional retinal detachment. PPV, epiretinal membrane peeling, retina attached with decaline, panretinal photocoagulation, and silicon oil injection were performed. Hypotony and then phthisis developed postoperatively in this eye.

## DISCUSSION

Combined cataract extraction and PPV remains controversial. Studies show that many surgeons prefer cataract extraction at the time of PPV even if the cataract is not clinically significant [7]. This is because cataract development in the phakic eye after PPV is common. If intraocular tamponade is used in conjunction with PPV, the rate increases; in case of silicone oil injection, the rate is reported to be as high as 100% [8]. Combined surgery is preferred because of its many advantages which are postoperative recovery time is shorter, visualization of the posterior pole is good during vitrectomy. The technique that combines phacoemulsification with PPV through a small limbal incision minimizes corneal distortion and gains better visual results [9]. However, there are possible disadvantages such as difficulty in visualizing the capsulorhexis because of an absent or reduced red reflex, cataract wound dehiscence caused by globe manipulation during vitreous surgery, intraoperative miosis after cataract extraction, bleeding from anterior structures, loss of corneal transparency from corneal edema, IOL decentration and iris capture in eyes with gas-air or silicone oil tamponade, and prismatic effects and undesirable light reflexes during vitreoretinal surgery [10]. Careful patient selection is important for the success of combined surgery. Phacoemulsification is most easily performed in eyes with mild to moderately dense nuclear sclerosis. Harder lenses may require excessive ultrasonic energy, much lens manipulation and prolonged irrigation, all of which contribute to intraoperative corneal edema [9].

Combined surgery in most cases is safe and allows for early visual rehabilitation [11]. In our experience, clear corneal phacoemulsification can be safely combined with PPV. The phacoemulsification technique is rapid and does not increase operating time significantly. Fundus visualization was achieved, especially when working at the retinal periphery. Because the corneal incision is made in avascular tissue, there is no bleeding into the anterior chamber and minimal postoperative inflammatory reaction [12]. The procedure does not interfere with vitreoretinal surgery. The incision is resistant to increased intra ocular pressure and globe manipulation during vitreoretinal surgery, even if gas or silicone oil injections are performed [12-14]. Scleral depression was also used successfully despite the IOL, and small cataract wound incision was sutured. Our results indicate that excellent functional visual acuity may be attained by combining PPV with phacoemulsification with posterior chamber IOL implantation. Postoperatively, visual acuity improved in 65 eyes (86.7%), was unchanged in 9 eyes (12.0%), and decreased in 1 eye (1.3%). 0.1≤ or better visual acuity was achieved in 44 (69.8%) eyes. Poor postoperative visual acuity (<0.1) was attributed to diabetic macular edema, retinal ischemia, or optic atrophy. Six eyes (8.0%) experienced an iatrogenic break that was successfully repaired during surgery.

The choice of IOL material is important when phacoemulsification is combined with vitreoretinal surgery. Because of the firm adhesion of silicone oil, and the condensation effects on silicone IOLs, silicone IOLs must be avoided in combined operations. Instead, a PMMA or acrylic polymer IOL should be used. In this study, we used acrylic polymer IOL in all cases [15]. It is suggested that IOL implantation should be delayed until the vitrectomy is completed or until the gas or silicone oil tamponade is performed. That is why, some surgeons believe that this maintains a small self-sealing incision and avoids light reflexes and prismatic effects from the IOL that might complicate visualization of the posterior pole, especially the most peripheral retina. However, IOL implantation before vitrectomy may be beneficial. The IOL enables the posterior capsule to be visualized, which reduces the risk for damaging the capsule with the vitreous cutter. IOL implantation in the presence of vitreous support is less complicated before vitrectomy. In addition, the IOL stabilizes the iris-capsule diaphragm and prevents posterior capsule bulging when endotamponades are used. Removal of epithelial cells from the lens capsule was performed to maintain good intraoperative visualization of the most peripheral part of the retina [1,15]. In this study, IOL implantation was performed before vitrectomy, and the posterior capsule remained intact in each case after surgery.

The recognition of true posterior vitreous is clinically important. The surgical implications of posterior hyaloid removal are obvious. Failure to recognize true posterior vitreous hyaloid may contribute to continued morbidity in patients after vitrectomy. If the residual vitreous cortex on the retina is left untreated during vitrecromy, this tissue may serve as a scaffold for continued neovascular proliferation, and may also contribute to persistent tangential tractional force on the retinal surface [5,6,17]. Triamcinolone acetonide is a water-insoluble steroid and has strong anti-inflamatory and antiangiogenic activity. TA-assisted vitrectomy increases the ability to achieve the anatomic goal of removal of cortical vitreous, and may decrease the blood-ocular break-down after surgery [17,18]. The use of intravitreal steroids have possible adverse effects such as glaucoma. As it has been found in this study, the use of TA enables us to see the residual vitreous cortex clearly and to treat it very easily. Therefore, we find this method to be helpful and beneficial for surgeons who perform a vitrectomy, especially for treating vitreoretinal interface abnormalities. In this study, In 6 eyes (8.0%), IOP increased temporarily for a few days after surgery; all were controlled with topical antiglaucomatous medication. No significant postoperative inflammation and fibrinous reaction occurred in all patients. Eight eyes (10.7%) had a mild iritis but which was controlled and resolved with use of topical steroids in a few days.

The present study is limited by its retrospective nature, non-randomized design, and heterogeneity in diagnoses of cases. However, the results of combined phacoemulsification, PPV, and IOL implantation show that visual acuity outcomes are generally favorable and complications are acceptable. We consider that this is a safe and effective method for combined surgery in cases with anterior and posterior segment pathology. Visual outcomes and complications depend primarily on the underlying posterior segment pathology. The use of intravitreal TA may simplify surgery especially posterior hyaloid removal and decreases the postoperative inflammation.

## Figures and Tables

**Table 1.** Distribution of Best Corrected Visual Acuity

Best Corrected Visual Acuity	Preoperative	Postoperative
0.5≥	-	15 (20.0%)
0.1-0.4	6 (8.0%)	38 (50.6%)
Counting fingers	22 (29.3%)	17 (22.7%)
Hand motions	47 (62.7%)	4 (5.4%)
Light perception (-)	-	1 (1.3%)

**Table 2.** Intraoperative and Postoperative Complications.

Complications	n	%
Mild iritis	8	10.7
Recurrent Vitreous hemorrhage	8	10.7
Posterior capsule opacification	7	9.3
Transient increased Intraocular pressure	6	8.0
Iatrogenic retinal tear	6	8.0
Postoperative corneal edema	5	6.7
Epiretinal membrane	3	4.0
Rubeosis iridis	3	4.0
Retinal detachment	2	2.7
Hyphema	2	2.7
Macular hole	1	1.3
Phthisis	1	1.3
